# Cognitive Change Questionnaire as a method for cognitive impairment screening

**DOI:** 10.1590/1980-57642015DN93000005

**Published:** 2015

**Authors:** Antonio Eduardo Damin, Ricardo Nitrini, Sonia Maria Dozzi Brucki

**Affiliations:** 1 MD, PhD, Behavioral and Cognitive Neurology Unit, Department of Neurology, School of Medicine, University of São Paulo (FMUSP), São Paulo SP, Brazil.; 2 Cognitive Disorders Reference Center (CEREDIC), School of Medicine-University of São Paulo (FMUSP), São Paulo SP, Brazil.

**Keywords:** dementia, cognitive assessment, diagnosis, mild cognitive impairment

## Abstract

The Cognitive Change Questionnaire (CCQ) was created as an effective measure of cognitive change that is easy to use and suitable for application in Brazil.

**Objective:**

To evaluate whether the CCQ can accurately distinguish normal subjects from individuals with Mild Cognitive Impairment (MCI) and/or early stage dementia and to develop a briefer questionnaire, based on the original 22-item CCQ (CCQ22), that contains fewer questions.

**Methods:**

A total of 123 individuals were evaluated: 42 healthy controls, 40 patients with MCI and 41 with mild dementia. The evaluation was performed using cognitive tests based on individual performance and on questionnaires administered to informants. The CCQ22 was created based on a selection of questions that experts deemed useful in screening for early stage dementia.

**Results:**

The CCQ22 showed good accuracy for distinguishing between the groups. Statistical models selected the eight questions with the greatest power to discriminate between the groups. The AUC ROC corresponding to the final version of the 8-item CCQ (CCQ8), demonstrated good accuracy in differentiating between groups, good correlation with the final diagnosis (r=0.861) and adequate internal consistency (Cronbach's α=0.876).

**Conclusion:**

The CCQ8 can be used to accurately differentiate between normal subjects and individuals with cognitive impairment, constituting a brief and appropriate instrument for cognitive screening.

## Abstract (pt)

O Questionário de Mudanças Cognitivas (QMC) foi criado para ser uma medida efetiva de mudança cognitiva que seja de uso fácil e adequado para o Brasil.

**Objetivo:**

Avaliar se o QMC pode distinguir com boa acurácia indivíduos normais daqueles com comprometimento cognitive leve (CCL) e/ou com estágio leve de demência e desenvolver a partir de um QMC inicial, com 22 questões (QMC22), um questionário com um menor número de questões.

**Métodos:**

Foram avaliados 123 indivíduos: 42 controles, 40 com CCL e 41 com demência leve. A avaliação foi realizada quanto ao desempenho em testes cognitivos e questionários aplicados aos acompanhantes. O QMC22 foi criado a partir de uma seleção de questões que especialistas achavam possuir acurácia na distinção entre indivíduos normais e aqueles com CCL ou demência leve.

**Resultados:**

O QMC22 mostrou boa acurácia na distinção dos grupos. Modelos estatísticos selecionaram as oito questões com o maior poder de discriminação entre os grupos. A area sob a curva –ROC correspondente à versao final do QMC8 demonstrou boa acurácia na distinção entre os grupos, boa correlação com o dignóstico final (r=0.861) e adequada consistência interna (Cronbach's α=0.876).

**Conclusão:**

O QMC8 pode ser usado na diferenciação entre indivíduos normais e aqueles com comprometimento cognitivo, é um instrumento rápido e apropriado de rastreio cognitivo.

## INTRODUCTION

Population aging has a significant impact on healthcare expenditures of developing countries. As populations age, the number of cases of dementia rises, representing a major cause of disability and mortality. A meta-analysis of Brazilian data from epidemiological studies has revealed a dementia prevalence of 5.1 to 19% among subjects aged 65 years or older.^1^

Despite the steady increase in the number of dementia cases, screening for dementia is not yet part of routine medical practice, reflected by the large number of dementia patients who have not been officially diagnosed. Routine clinical evaluation using medical history and physical examination is insufficient for detecting dementia where over 50% of individuals with dementia do not receive a diagnosis of dementia from their general practitioner.^2^ In Brazil, a study performed in a tertiary general practice clinic showed that only 16% of elderly individuals with dementia were correctly diagnosed by their general practitioner.^3^

In clinical practice, there is a need for a screening instrument sufficiently sensitive to detect the early stages of dementia, for which tests such as the MMSE (Mini-Mental State Examination) alone may be insensitive, especially among individuals with high educational level.^4^

Cognitive assessments administered to the informant provide collateral sources of information and evaluate the changes occurring over time in an individual's ability to perform everyday tasks. Compared with physician-administered performance tests, informant-administered assessments are also less subject to the influence of sociocultural differences and education and may be more sensitive than performance tests for the early detection of dementia.^5-7^

Based on information gathered from informants, Galvin et al. (2005)^5^ devised an 8-item questionnaire called the AD8. Scores ≥2 on the questionnaire indicate good accuracy for discriminating among patients with CDRs of between 0 and 0.5.^5-7^

The Cognitive Change Questionnaire (CCQ) was developed to screen for the early stages of dementia in Brazilian patients, drawing on the AD8, as a short screening tool to detect cognitive impairment. Based on AD8 questions, and other questions more suited for use in the Brazilian population, the first 22-item version of the CCQ was created. The initial reason for the development of the CCQ was the creation of a cognitive test that can be applied to the informant. Specialists with extensive expertise in cognition selected the questions comprising the CCQ based on their personal assessment in clinical practice with mild dementia patients. The aims of this study were to evaluate whether application of the Cognitive Change Questionnaire (CCQ) could adequately distinguish normal subjects from those with mild cognitive impairment (MCI) and/or early stage dementia and to compare this accuracy with performance-based or informant-administered tests previously validated for use in detecting dementia among Brazilian patients. The specific objectives were to develop a final model test based on the CCQ22 but containing only eight questions (CCQ8) for use in clinical practice as an instrument for cognitive screening instrument, and to assess the CCQ8's accuracy in identifying individuals with MCI or early stage dementia.

## METHODS

We performed a prospective clinical study from April 2007 to September 2010, approved by the Research Ethics Committee of the Hospital das Clínicas, University of São Paulo (HC/FMUSP). A total of 123 subjects were evaluated: 42 controls, 40 patients with mild cognitive impairment (MCI) and 41 with mild dementia. Cases included for evaluation were randomly assigned in the consecutive order that they were referred for assessment of cognitive complaints to the Reference Center for Cognitive Disorders (CEREDIC). Control subjects were individuals with no changes on cognitive performance tests and without functional impairment, comprising patient family members and subjects living in the community. Patients with dementia were defined according to the DSM-IV^8^ while Alzheimer's disease patients were diagnosed by the NINCDS-ADRDA criteria.^9^ The Clinical Dementia Rating Scale (CDR) defined the severity of dementia and patients with CDR of 1 were included.^10^ Individuals with MCI were defined by the criteria of the European Consortium on Alzheimer's Disease.^11^ Patients were excluded if they had no informant, presented visual or auditory deficits precluding performance of the cognitive assessments, had subjects or caregivers who refused to participate or give informed consent, and if they suffered from moderate to severe stages of dementia (CDR=2 and 3).

All subjects and informants received explanations about the study and provided informed consent.

The sample size was calculated in the final phase of the study based on patient scores on the CCQ22 and the ability of this assessment to differentiate among groups (control, MCI and dementia), considered adequate for the purposes of this study.

All subjects underwent clinical evaluation with complete physical and neurological examination. Participants were evaluated by the Mini-Mental State Examination (MMSE),^12,13^ CAMCOG,^14^ CDR^10^ and the brief cognitive screening battery.^15^ Patient informants were interviewed and completed the Functional Activities Questionnaire (FAQ),^16^ the IQCODE,^17^ CDR,^10^ Neuropsychiatric Inventory (NPI)^18,19^ and the 22-item CCQ.

The CCQ contained 22 questions extracted from tests administered to informants (Box 1). These questions were previously used in clinical practice as items on tests such as the AD8, FAQ, IQCODE, activities of daily living,^20^ Index of ADL^21^ and the Blessed Dementia Scale.^22^ In addition to this selection, questions 16 and 21 were included for complementation and questions 10 and 22 were adapted from their original form to be more relevant for use in Brazil.

Informants were asked to answer the questions on the CCQ22 while considering any changes over time in the patient. For each question, the informants were presented with the options "yes" (yes, there was a change from a previous level) or "no" (no change). After completion of the test, the answers were scored by attributing one point for each yes answer.

The final diagnosis was used as the gold standard for the classification of subjects. Diagnoses were established by consensus of a panel of experts in the area of cognitive science who belong to the CEREDIC, and were based on the results of imaging and laboratory tests, neurological and cognitive evaluations as well as questionnaires administered to informants. In order to standardize diagnoses, the same members of the panel remained throughout the study and were blinded to the CCQ22 results.

**Statistical analysis.** SPSS (Statistical Package for the Social Sciences) version 16.0 and STATISTICA version 9 were used for statistical analysis and comparisons among the three groups (control, MCI and dementia). ANOVA was used for parametric data and the Kruskal-Wallis test was used for nonparametric data with post hoc analysis using Dunn's test. A value of p <0.05indicated statistical significance. Spearman`s correlation coefficient evaluated the correlations between tests. The ROC (Receiver Operating Characteristics), and its coordinates, were used to analyze the performance, accuracy and cut-off scores of the tests. The internal consistency of the CCQ8 (final model) and CCQ22 was assessed by Cronbach's alpha and values above 0.7 indicated adequate internal consistency.^23^

## RESULTS

The etiology of dementia in this sample was as follows: 20 subjects (47%) were diagnosed with AD, 9 (22%) with vascular dementia (VD), 3 (7%) with AD associated with cerebrovascular disease and 24% with other dementias. The subtypes of MCI were classified as follows: 21 (52%) as amnestic multiple-domains, 9 (22%) as amnestic single-domain and the remainder as non-amnestic.

Table 1 shows the descriptive statistics of the groups. Age was assessed by ANOVA. The Kruskal-Wallis Test assessed the other variables, as they were not suitable for analysis by parametric tests. There was no significant difference in the ratio of men to women between groups based on the Chi-square test (p=0.223). Post-hoc analysis of the Kruskal-Wallis tests using Dunn's test to compare groups (control, MCI and dementia) two by two showed significant differences in the scores on all tests, including the CCQ22, with the exception of scores on the NPI between the MCI and dementia groups.

**Table 1:** Descriptive analysis of the groups (control, MCI, dementia).

Variable	Group	Mean (SD)	Median value	p-value*	
Age (years)	Control	68.27 (7.34)	69	0.104	
	MCI	67.05 (9.80)	68	
	Dementia	71.24 (9.51)	74	
Education (years)	Control	7.48 (4.48)	6	0.06	
	MCI	6.49 (4.77)	4	
	Dementia	5.44 (4.32)	4	
MMSE	Control	28.39 (1.61)	29	<0.001	
	MCI	25.68 (3.62)	27	
	Dementia	19.07 (4.85)	19	
IQCODE	Control	2.96 (0.52)	3.06	<0.001	
	MCI	3.44 (0.68)	3.42	
	Dementia	4.35 (0.43)	4.5	
CAMCOG	Control	92.04 (10.95)	94	<0.001	
	MCI	78.84 (13.65)	83	
	Dementia	56.91 (18.73)	58	
CCQ22	Control	3.00 (2.65)	3	<0.001	
	MCI	10.95 (4.70)	11	
	Dementia	17.54 (3.56)	18	

* Kruskal-Wallis Non-parametric ANOVA. MMSE: Mini-Mental State Examination. IQCODE: Informant

Questionnaire on Cognitive Decline in the Elderly. CAMCOG: Cambridge Cognitive Examination. CCQ22: Cognitive Change Questionnaire - 22 item.

There were no statistically significant differences in age, educational level, gender, degree of kinship and living situation of the informant with the patient, among the control, MCI and dementia groups.

The statistical power of discrimination among the three groups (control, MCI and dementia) was evaluated for each of the questions on the CCQ22. A generalized linear model using a multinomial distribution and cumulative logit link function was used to identify the most discriminative questions. The Newton-Raphson method for robust estimation, and likelihood ratio tests were used to assess the significance of individual questions. Questions 1, 6, 8, 19, 20 and 21 were removed from the initial model because they demonstrated a low power of discrimination between groups. In the final model, the six questions that demonstrated the statistical power to discriminate between groups were questions 9, 10, 11, 13, 18 and 22. Comparisons were made between all possible combinations of the questions using analysis of covariance and multi-collinearity.

A final questionnaire was created by the addition of questions 4 and 5 to the six questions cited above as this combination showed the greatest Spearman correlation coefficient with the final diagnosis, and the largest area under the ROC curve for discrimination between control subjects and patients with cognitive impairment associated with MCI or dementia.

The final questionnaire (CCQ8) was established by examining clinical parameters based on the statistical analysis (Table 2). From the coordinates of the ROC curve, cut-off scores were obtained for the CCQ8 and the diagnostic properties of the questionnaire are shown in Table 3. The CCQ8 was also compared to a model that contained only the six initial questions (CCQ6), but the diagnostic ability of the CCQ6 proved lower than the CCQ8.

**Table 2:** Final Model of the CCQ8 (Portuguese version in Box 1).

Was there a change in recent years in the items listed below?	Yes	No
1.Difficulty in learning how to use a tool, appliance or gadget (such as a computer, microwave, remote control)		
2.Forgetting the correct month or year		
3. Difficulty in using the phone to make calls		
4. Difficulty in using a car, bus, taxi or boat alone		
5. Difficulty in taking medications without supervision		
6.Trouble keeping updated with important information concerning the community or country		
7. Difficulty in expressing their own opinions regarding family issues		
8.Difficulty going out for a walk alone and returning home without getting lost		
Total		

**Table 3:** Diagnostic properties of the CCQ8 among groups.

CCQ8	Cut-off	Sensitivity	Specificity	PPV*	NPV#	Accuracy
C × MCI	>1	97.6 %	66.7%	78.4%	95.6%	83.8%
	≥2	78.0%	93.9%	94.1%	77.5%	85.1%
C × Dem.	≥4	97.5%	100%	100%	97.0%	98.6%
MCI × Dem.	≥4	97.5%	83.9%	72.2%	96.3%	80.2%
C × CI	≥1	98.7%	66.6%	87.9%	95.6%	89.5%
	≥2	88.9%	93.9%	97.3%	77.5%	90.3%

* PPV: positive predictive value.

# NPV: negative predictive value. C: Control group. MCI: mild cognitive impairment group. Dem: dementia group. CI: cognitive impairment group (MCI and Dementia).

The mean scores on the CCQ8 were 0.39 (95% CI: 0.21-0.87) in the control group, 3.17 (95% CI: 3.01-4.64) in the MCI group and 6.50 (95 % CI: 6.01-7.52) in the dementia group. These results differed significantly according to the Kruskal-Wallis test and post-hoc analysis by Dunn's test.

Each group was divided into three subgroups based on educational level (0-4 years, 5-8 years and greater than 8 years), and there were no significant differences in CCQ8 scores among the different educational level groups.

Table 4 shows Spearman correlation coefficients of all the instruments used in this study for the CCQ22, the CCQ8 and the final diagnosis (gold standard). Table 5 shows the area under the ROC curve for all tests in the study. Values in bold indicate those tests that had the highest accuracy in each of the groups. Cronbach's alpha was 0.876 for the CCQ8 and 0.936 for the CCQ22.

**Table 4:** Spearman correlation coefficients (r) among variables and CCQ8, CCQ22 and final consensus diagnosis.

	CCQ8	CCQ22	Final diagnosis
Age	0.012	0.035	0.151
Gender	0.153	0.137	0.137
Education	–0.244*	–0.248*	–0.229*
CCQ8	1.000	0.945*	0.861*
CCQ22	0.945*	1.000	0.853*
MMSE	–0.679*	–0.665*	–0.737*
CAMCOG	–0.653*	–0.646*	–0.725*
CDR	0.873*	0.859*	0.916*
FAQ	0.858*	0.872*	0.823*
IQCODE	0.769*	0.824*	0.751*
NPI	0.479*	0.475*	0.348*

* p<0.05 (Two-tailed analysis). MMSE: Mini-Mental State Examination, CAMCOG: Cambridge Cognitive Examination; IQCODE: Informant Questionnaire on Cognitive Decline in the Elderly; FAQ: Functional Activities Questionnaire; NPI: Neuropsychiatric Inventory; CCQ22: cognitive change questionnaire - 22 item; CCQ8: cognitive change questionnaire - 8 item.

**Table 5:** Area under the ROC curve (AUC) for all tests among groups.

	C × MCI	C × Dementia	MCI × Dementia	C × CI
CCQ8	0.938*	0.999*	0.892*	0.968*
CCQ22	0.934*	0.999*	0.895*	0.966*
CDR	0.902*	1.000*	0.934*	0.951*
FAQ	0.839*	0.998*	0.917*	0.917*
IQCODE	0.742*	0.987*	0.882*	0.864*
NPI	0.809*	0.782*	0.519	0.795*
MMSE	0.760*	0.971*	0.860*	0.865*
CAMCOG	0.844*	0.952*	0.833*	0.896*

* p<0.001. MMSE: Mini-Mental State Examination. CAMCOG: Cambridge Cognitive Examination. IQCODE: Informant Questionnaire on Cognitive Decline in the Elderly. FAQ: Functional Activities Questionnaire. NPI: Neuropsychiatric Inventory. CCQ22: cognitive change questionnaire - 22 item. CCQ8: cognitive change questionnaire - 8 item.

## DISCUSSION

The CCQ22 and final model of the CCQ8 were highly accurate in distinguishing between control subjects and patients with MCI or early stage dementia. The lack of accurate, fast and easily administered tools for cognitive screening adapted for use in Brazil is one of the many reasons for the large number of individuals with dementia who have not been formally diagnosed.

Control, MCI and dementia groups were homogeneous in terms of age, gender and educational level. The scores from all of the tests used in the study, including the CCQ22 and CCQ8, showed significant differences between means for the control, MCI and dementia groups, except for the NPI test, which showed no significant difference in scores between the MCI and dementia groups.

We concluded that the neuropsychiatric disorders began early, as the MCI group had more events compared with the control subjects, in agreement with the report of Geda et al.^24^ Despite this result, the frequency and severity of neuropsychiatric symptoms were not greater in the mild dementia stage as compared with MCI.

In general, the tests given to the informants had higher correlations with the final diagnosis than tests based on individual performance. We concluded, in agreement with other studies, that performance tests may have lower accuracy for diagnosing subjects with early stage dementia. This inaccuracy indicates that these tests are inadequate for cognitive screening.^4,5^

Among tests given to informants, the IQCODE had the lowest accuracy across all of the groups, reinforcing findings of studies showing that the IQCODE is not a high-accuracy instrument for discrimination of groups.^25-27^

On assessments of diagnostic accuracy based on the area under the ROC curve, the CCQ22 and the CCQ8 were the most accurate tests across all groups. The AD8 translated to Brazilian-Portuguese exhibited high reliability (alpha=0.818) and good accuracy between CDR=0 and > 0 (AUC:0.861) and CDR=0 and CDR=0.5 (AUC:0.769)^28^ albeit lower levels of accuracy compared to those attained by the CCQ8 version developed.

With regards to informants, the three groups were homogeneous in gender, educational level, age, degree of kinship and whether they lived with the subject.

Unlike the MMSE, the results of the CCQ8 were unaffected by educational level. Thus, the cut-off scores of the CCQ8 did not need to be adapted to control for differences in educational levels, facilitating their use in the interpretation of the results on cognitive screening.

The CCQ8 showed the highest accuracy in distinguishing between subjects with normal cognition and those with cognitive impairment and can therefore be considered an essential screening tool. Moreover, as the CCQ8 is highly accurate in distinguishing between dementia and control groups and between MCI and dementia groups, it may be helpful toward defining the diagnosis of subjects with cognitive impairment, especially when combined with performance tests such as the MMSE. However, further studies are needed to confirm this hypothesis. Comparison of the coordinates of the ROC curves between controls and patients with cognitive impairment indicated that a score of ≥2 is suggestive of cognitive impairment while a score ≥4 is suggestive of dementia.

A cut-off score of ≥1, which has a sensitivity of 98.7% when detecting cognitive impairment based on the CCQ8, would be a more sensitive indicator of cognitive impairment, although the specificity would then be lower. This is congruent with the results of a recent study indicating that the AD8 had high sensitivity but very low specificity.^29^ Similarly, in a study performed in Japanese elders, sensitivity was 88.4%, and specificity 68.4%, for a score of 1/2.^30^

The CCQ8 had the second-highest correlation (r=0.861) where the CDR outperformed the CCQ8 in relation to final clinical diagnosis (r=0.916). However, the CDR was used in the assessment of functional impairment and is an extensive scale that may not be suitable for cognitive screening. The CCQ22 and CCQ8 showed significant correlations with performance on cognitive tests and with questionnaires administered to informants, previously validated for use in clinical practice. However, correlations were higher for questionnaires administered to informants.

In regards to internal consistency, the CCQ8 and the CCQ22 had Cronbach's alpha values of 0.876 and 0.936, respectively. Values greater than 0.7 indicate good internal consistency, whereas values greater than 0.9 are indicative of redundant items in a scale. Thus, the Cronbach's alpha value of the CCQ22 indicates that it contains redundant questions, whereas the CCQ8 does not.^23^

A possible criticism of the present study is that the assessments were performed in a specialized, tertiary clinical setting. Thus, the findings may not be representative of a community sample. Further studies using the questionnaires in larger population samples are required, particularly for the CCQ8.

The final model, CCQ8, is brief, with only eight items and is more accurate than the CCQ22, especially with respect to distinguishing between normal subjects and individuals with cognitive impairment, an essential function of a cognitive screening test. The next step towards adoption of the CCQ8 is its application in different population samples, including both tertiary centers and primary health care facilities, to confirm the findings of this study and validate its use as a tool for cognitive screening in Brazil.

A comparison of the accuracy of the CCQ22 and CCQ8 with validated tests used in clinical practice revealed that both CCQs have adequate accuracy, often higher than the performance tests and tests administered to informants in this study. Both versions have demonstrated very good correlation with the final diagnosis and with validated tests currently used in the clinical setting.

In conclusion, the results show that the CCQ8 is a good cognitive screening tool for diagnosis of patients with cognitive impairments based on the following features:

- It is a brief questionnaire with few questions, facilitating rapid application and easy interpretation;
- It is well correlated with the final diagnosis and with the cognitive and functional tests currently used and validated in the field;
- It is not influenced by educational level;
- It had one of the highest accuracies of all analyzed tests and high sensitivity and specificity;
- It is a single questionnaire designed in Brazil based on a sample of the Brazilian population (although not a community sample);
- It has good positive and negative predictive ability;
- It is internally consistent.

Further studies involving this instrument in clinical settings are necessary to replicate these results among other samples.

## Box 1

In portuguese. Highlighted questions comprised the CCQ8.

**Table:**

**AD22**				
Favor preencher os itens abaixo, marcando um x abaixo no local que achar mais apropriado. Atenção: “sim, uma mudança (uma alteração)” indica que você pensa que tem havido mudança (alteração) nos últimos anos causada por problemas cognitivos (pensamento e memória).

**Table:**

		Sim, uma mudança	Não, nenhuma	N/A (não se aplica) ou não sei
1.	Problemas de julgamento (p. ex: cair em contos do vigário, más decisões financeiras, comprar presentes inadequados para os que os recebem).			
2.	Interesse reduzido em hobbies/atividades			
3.	Repete perguntas, estórias, afirmações			
4.	Dificuldade para aprender como usar um instrumento, eletrodoméstico ou outro aparelho (engenhoca) (p. ex.: vídeo-cassete, computador, microondas, controle remoto).			
5.	Esquece o mês ou o ano correto			
6.	Dificuldade para lidar com assuntos financeiros complicados (p. ex: controle do saldo no talão de cheques, imposto de renda, pagamento de contas).			
7.	Dificuldade para lembrar-se de compromissos			
8.	Problemas constantes com pensamento e/ou memória			
9.	Dificuldade para usar o telefone para fazer ligações			
10.	Dificuldade para usar carro, ônibus, táxi ou barco sozinho.			
11.	Dificuldade para tomar remédios sem supervisão			
12.	Dificuldade para compreender uma notícia ou um filme ou programa de rádio ou televisão			
13.	Dificuldade para se manter atualizado sobre os fatos importantes da comunidade ou do país			
14.	Dificuldade para se lembrar do que conversou nos últimos dias			
15.	Dificuldade para lembrar-se onde as coisas são guardadas usualmente			
16.	Dificuldade para contar o que acabou de ver ou ouvir			
17.	Dificuldade para lembrar uma mensagem			
18.	Dificuldade para expressar opiniões próprias sobre assuntos de família			
19.	Dificuldade para terminar alguma coisa começada			
20.	Dificuldade para lidar com pequenas somas de dinheiro			
21.	Dificuldade para tomar parte em uma conversa			
22.	Dificuldade para sair para uma caminhada sozinho e voltar para casa sem se perder			
Total

**Table:**

O AD22 é entregue ao informante sobre uma prancheta e geralmente leva menos do que 3 minutos para ser completado. Itens classificados como “sim, uma mudança (uma alteração)” são somados para produzir o escore total do AD22.				
